# Human umbilical cord mesenchymal stem cells ameliorate erectile dysfunction in rats with diabetes mellitus through the attenuation of ferroptosis

**DOI:** 10.1186/s13287-022-03147-w

**Published:** 2022-09-05

**Authors:** Huan Feng, Qi Liu, Zhiyao Deng, Hao Li, Huajie Zhang, Jingyu Song, Xiaming Liu, Jihong Liu, Bo Wen, Tao Wang

**Affiliations:** 1 Department of Urology, Institute of Urology, Tongji Hospital, Tongji Medical College, Huazhong University of Science and Technology, Wuhan, Hubei China; 2 Department of Urology, Shenzhen Hospital of Integrated Traditional Chinese and Western Medicine, Shenzhen, Guangdong China; 3 Shenzhen Huazhong University of Science and Technology Research Institute, Shenzhen, Guangdong China

**Keywords:** Erectile dysfunction, Human umbilical cord mesenchymal stem cells, Type 1 diabetes mellitus, Type 2 diabetes mellitus, Ferroptosis

## Abstract

**Background:**

Erectile dysfunction (ED), as one of the most prevalent consequences in male diabetic patients, has a serious impact on men's physical and mental health, and the treatment effect of diabetic mellitus erectile dysfunction (DMED) is often worse. Therefore, the development of a novel therapeutic approach is urgent. As stem cells with high differentiation potential, human umbilical cord mesenchymal stem cells (HUCMSCs) have been widely used in the treatment of diseases in other systems, and are expected to be a promising strategy for the treatment of DMED. In this study, we investigated the role of HUCMSCs in managing erectile function in rat models of type 1 diabetes mellitus (T1DM) and type 2 diabetes mellitus (T2DM) and compared the effects of two different injection methods.

**Methods:**

T1DM and T2DM ED rats were given labelled HUCMSCs by corpus cavernosum injection and tail vein injection, respectively. ICP and MAP were monitored simultaneously by electrical stimulation four weeks after injection to indicate the erectile function of rats. To track the development and colonisation capabilities of stem cells, we performed EdU assay with penile tissue. The histological changes of the penis were observed by hematoxylin–eosin staining, and Masson’s trichrome staining was conducted to evaluate the smooth muscle content and the degree of fibrosis in the rat penis. Then, we employed specific kits to measure the level of NO, cGMP, MDA, SOD and Fe in penis. Electron transmission microscopy was implemented to observe morphology of mitochondria. Besides, western blot and immunofluorescence staining were performed to demonstrate the expression of ferroptosis-related genes.

**Results:**

We found that HUCMSCs improved erectile function in T1DM and T2DM ED rats, with no difference in efficacy between corpus cavernosum injection and tail vein injection. The EdU assay revealed that only a tiny percentage of HUCMSCs colonised the corpus cavernosum, while smooth muscle in the penis expanded and collagen decreased following HUCMSC injection. Moreover, the levels of oxidative stress in the penis of the rats given HUCMSCs were dramatically reduced, as was the tissue iron content. HUCMSCs normalised mitochondrial morphology within corpus cavernosum smooth muscle cells (CCSMCs), which were characteristically altered by high glucose. Furthermore, the expression of ferroptosis inhibitory genes SLC7A11 and GPX4 was obviously elevated in CCSMCs after stem cell management, but the abundances of ACSL4, LPCAT3 and ALOX15 showed the polar opposite tendency.

**Conclusions:**

HUCMSCs can effectively and safely alleviate erectile dysfunction in T1DM and T2DM ED rats, while restoring erectile function by attenuating diabetes-induced ferroptosis in CCSMCs. Additionally, this study provides significant evidence for the development of HUCMSCs as a viable therapeutic strategy for DMED.

**Supplementary Information:**

The online version contains supplementary material available at 10.1186/s13287-022-03147-w.

## Introduction

Erectile dysfunction (ED) is one of the most common male sexual dysfunction diseases, with 150 million men suffering from varied degrees of ED around the world [1]. As one of the major complications in men with diabetes mellitus (DM), the incidence of ED in diabetic patients is up to 75% [2]. Men with DM tend to suffer from ED about 10 years earlier than the general population [3]. By 2045, it is estimated that there will be approximately 700 million individuals living with diabetes globally [4]. Despite the fact that PDE5i is now widely acknowledged as a first-line treatment for ED, up to 35% of patients do not react to it [5], which might be owing to diabetes and the consequent severe neurological or vascular lesions. As a result, a more effective therapy for diabetes mellitus erectile dysfunction (DMED) is required.

Mesenchymal stem cells are generated from human bone marrow, umbilical cord tissue, adipose tissue, lung tissue, tooth pulp, and placenta and have immune control, regeneration, and pluripotent differentiation capabilities [6, 7]. Multiple studies have proven that mesenchymal stem cells may be utilised to treat corticosteroid-resistant graft-versus-host disease, multiple sclerosis, osteoarthritis, heart failure, and other indications without substantial adverse effects [8–11].

Human umbilical cord mesenchymal stem cells (HUCMSCs) have a number of benefits over stem cells generated from other organs. To begin with, the collection of HUCMSCs is non-invasive, which reduces the risk of infection significantly. HUCMSCs, on the other hand, have a greater proliferation capacity and can sustain steady growth after the tenth passage, suggesting that they might theoretically have better therapeutic effectiveness at the same dosage [12]. Second, due to their distinct gene expression profile, HUCMSCs are barely tumorigenic and have a higher differentiation capability and self-renewal potential [13, 14]. Low expression of major histocompatibility complex (MHC) class I molecules, a significant absence of MHC class II molecules, and a lack of co-stimulatory ligands such as CD40, CD80, and CD86 further reduced the immunogenicity of HUCMSCs [15]. As a result, it's clear that HUCMSCs might be an excellent therapy option.

Some clinical trials have indicated that HUCMSCs can safely and effectively treat lung damage and improve decreased lung function in patients with COVID-19 in recent years [16, 17]. HUCMSCs also improved in situ healing capabilities in rhesus monkeys with severe uterine adhesions and decreased mortality in rats with ARDS accompanied by sepsis, according to two other preclinical investigations [18, 19].

Ferroptosis, first proposed by Dixon et al. in 2012 [20], was identified as a non-apoptotic programmed death pathway that relies on iron ions and reactive oxygen species (ROS). Ferroptosis is distinctly different from apoptosis, necrosis, pyroptosis, and autophagy, and is characterised by iron accumulation [21] and overproduction of lipid peroxides [22]. Meanwhile, the characteristic morphological changes of mitochondria in ferroptotic cells can be observed under transmission electron microscope (TEM) [23]. Abundant evidence indicates ferroptosis is involved in various neurodegenerative diseases, and abnormal accumulation of iron in brain tissue will induce massive ROS production in brain cells [24–26]. Liu et al. demonstrated that puerarin prevented pressure overload-induced heart failure by attenuating ferroptosis [27]. In addition, ferroptosis also plays an important role in hepatocellular carcinoma [28–31], colorectal cancer [32], melanoma [33] and becomes a promising therapeutic target of anti-cancer drugs. However, whether ferroptosis is involved in the progression of DMED remains unknown.

Based on these findings, we created animal models of T1DM and T2DM ED rats, with the goal of determining the possible therapeutic benefits of HUCMSCs on DMED and investigating the underlying processes.

## Materials and methods

### Cell identification and culture

HUCMSCs were donated by Shandong Qilu Cell Therapy Technology Co., Ltd and suspended in specialised medium (Dulbecco’s modified Eagle’s medium [DMEM, Gibco, USA] with supplements for HUCMSCs [Qilu Cell Therapy Technology, China]) and cultivated at 37 °C in a 5% CO_2_ incubator. The fourth passage HUCMSCs were identified by flow cytometry. Flow cytometry was used to identify the HUCMSCs in the fourth passage. For surface marker verification, the following antibodies were used: CD29, CD31, CD34, CD45, CD90, and CD105 (BD Biosciences, USA).

The multilineage differentiation potential of HUCMSCs was examined using third passage cells, as previously described. For 14 days, cells were grown in adipogenic medium (DMEM containing 10% FBS, 2 mM dexamethasone, 2 mg/L insulin, 0.5 mM 3-isobutyl-1-methylxanthine, 0.2 mM indomethacin) and for 28 days in osteogenic medium (DMEM containing 10% FBS, 2 mM dexamethasone, 1 M sodium glycerol phosphate, 10 mM vitamin C). The capacity to develop into adipocytes and osteoblasts was then assessed using Oil red O and Alizarin red S staining.

Our team's typical approach involved isolating corpus cavernosum smooth muscle cells (CCSMCs) from rat penis and purifying them using a differential adhesion method over the course of two weeks [34]. The CCSMCs were then grown in DMEM with 10% FBS added. CCSMCs were detected by immunofluorescence using antibodies against α-smooth muscle actin (α-SMA; 1:200; Affinity Biosciences, AF1032, USA) and desmin (1:200; Affinity Biosciences, AF5334, USA).

The co-culture system was constructed by a polycarbonate membrane of Costar Transwell Inserts (Corning, No. 3412, USA). The upper and lower chambers were seeded with CCSMCs and HUCMSCs, respectively. The ratio of CCSMCs to HUCMSCs was 1:1.

### Experiments with animals

Thirty 7-week-old male Sprague–Dawley (SD) rats and thirty 7-week-old male Zucker Diabetic Fatty (ZDF) rats were obtained from Beijing Vital River Laboratory Animal Technology Co., Ltd., and they were all fed adaptively for 7 days after fasting overnight. For type 1 diabetes mellitus (T1DM), streptozotocin (STZ; 60 mg/kg; Sigma-Aldrich) was diluted in citrate phosphate buffer (50 mM; pH 4.5) and injected intraperitoneally into SD rats. The rest 6 SD rats were only administrated with the citrate phosphate buffer and placed in the control group. The blood glucose levels of all SD rats were measured three and seven days later, and rats with fasting blood glucose > 16.7 mmol/L at both tests were classified as T1DM. Purina 5008 was given to ZDF rats for 4 weeks to induce type 2 diabetic mellitus (T2DM). The remaining seven rats were designated as the control group and continued to be fed with standard chow. The blood glucose levels of ZDF rats were tested three- and seven-days following induction feeding, and animals with fasting blood glucose levels > 16.7 mmol/L at both assessments were defined as T2DM.

Ten weeks later, all of DM rats survived. Following that, an apomorphine (APO) test was performed to identify the rats with DMED, and those with a negative erection were classified as DMED rats, as in the prior study [35]. Through the APO test, 18 rats each were diagnosed with T1DM and T2DM ED. SD rats were further separated into four groups (DMED group, *n* = 5; DMED + corpus cavernosum injection (CI) group, *n* = 7; DMED + tail vein injection (VI) group, *n* = 6; control group, *n* = 6). Meanwhile, ZDF rats were separated into four groups (DMED group, *n* = 5; DMED + corpus cavernosum injection (CI) group, *n* = 7; DMED + tail vein injection (VI) group, *n* = 6; control group, *n* = 7). Two of the groups (the DMED + CI group and DMED + VI group) received HUCMSCs injections at different sites and the injection dose was 1 * 10^6^ HUCMSCs per rat (injection volume 50 μL), whereas the DMED group and the control group received same volume of saline treatment. Moreover, HUCMSCs were labelled with 5-ethynyl-2’-deoxyuridine (EdU; Beyotime, China) according to the manufacturer’s instructions.

### Erectile function evaluation

The intracavernous pressure (ICP) was monitored four weeks following injection, as previously described [36]. Ketamine (100 mg/kg) and midazolam (5 mg/kg) were used to anaesthetize rats. To measure ICP, a 25-gauge needle was placed into the left penile crus. With micro scissors, a V-shaped incision was created in the carotid artery, a PE-50 tube was inserted, and it was ligated and fastened. The PE tube was pre-filled with heparinised saline (200 IU/mL) and attached to a signal collection device (AD Instruments, Australia), allowing for continuous monitoring of mean arterial pressure (MAP). After that, bipolar electrodes were used to electrically activate the cavernous nerve (15.0 Hz; 5.0 V; for 1 min). The ratio of maximum ICP (max ICP) to MAP (max ICP/MAP) was used to determine erectile function. The rats were slaughtered after their erectile function was assessed, and the penises were dissected from the original tissue. For histologic research, one third of the penis was preserved in paraffin, while the rest were kept at -80 °C for other experiments.

### Immunohistochemistry, hematoxylin–eosin (H&E) staining, and Masson's trichrome staining

Immunohistochemistry was performed as previously reported [37]. After antigen retrieval and dewaxed, sections of corpus cavernosum were incubated with antibodies against endothelial nitric oxide synthase (eNOS; 1:100; Affinity Biosciences, AF0096, USA) or neuronal nitric oxide synthase (nNOS; 1:100; Affinity Biosciences, AF6249, USA) antibodies, followed by biotinylated secondary antibodies.

The tissues were fixed for 24 h in 4% paraformaldehyde (Servicebio, China) before being imbedded in paraffin. Serial 4 μm paraffin-embedded slices were obtained and dewaxed in xylene I and xylene II for 10 min each, before being rehydrated in a variety of ethanol concentrations (100% for 5 min, 100% for 5 min, 95% 5 min, 90% 5 min, 80% 5 min, 70% 5 min). The slices were then washed three times in distilled water (5 min each). Finally, according to the manufacturer's instructions, sections were stained with H&E solution (Servicebio, China).

Masson’s trichrome staining was conducted according to the standard protocol. The area of smooth muscle and collagen was determined with ImageJ software. The ratio of smooth muscle to collagen was taken as an indicator of the degree of fibrosis in the corpus cavernosum.

### Fluorescence staining

A superoxide anion fluorescent probe (Dihydroethidium, DHE; Yeasen Biotechnology) and the Reactive Oxygen Species Assay Kit (2,7-Dichlorodi-hydrofluorescein diacetate, DCFH-DA; Yeasen Biotechnology) were used to measure the quantity of ROS in the corpus cavernosum. DHE and DCFH-DA were used to incubate tissue and cell sections, respectively.

As previously stated, penis samples were preprocessed. GPX4 (1:200; ABclonal, A1933, China) and ACSL4 (1:200; ABclonal, A1933, China) were used to incubate sections (1:200; Affinity Biosciences, DF12141, USA). The slides were then treated with the appropriate secondary antibodies before being stained with 4', 6-diamidino-2-phenylindole (DAPI, Invitrogen) for nuclear staining.

Sections were assessed for EdU labelling using the BeyoClick™ EdU Assay Kit with Alexa Fluor 647 (Beyotime, China), for 30 min at room temperature. The above sections were inspected under a fluorescence microscope.

### Measurement of oxidative activity

The levels of malondialdehyde (MDA) and superoxide dismutase (SOD) reflected the oxidative and antioxidant activities, respectively. The Micro MDA Assay Kit (Solarbio, BC0025, China) and SOD assay kit (Nanjing Jiancheng Bioengineering Institute, A001-3-2, China) were used to detect these two indicators according to the manufacturer’s instructions.

### Assessment of iron content

The content of Fe in corpus cavernosum was evaluated by Tissue Iron Assay Kit (Nanjing Jiancheng Bioengineering Institute, A039-2-1, China) according to the manufacturer’s instructions.

### Examination of nitric oxide (NO) and cyclic guanosine monophosphate (cGMP) levels

Nitrate-nitrite Assay Kit (Beyotime, S0024, China) was used to detect the level of NO, and the cGMP levels were determined ELISA kit (R&D Systems, F15182, USA) according to the standard protocol. The results were normalised to the protein concentration.

### Transmission electron microscope analysis

Cells were fixed with Electron Microscope Fixative Solution (Servicebio, G1102, China) for 1 h, then were re-fixed with 1% osmic acid in 0.1 M phosphate buffered saline (pH 7.4) at room temperature for 2 h. Next, adding 50%, 70%, 80%, 90%, 95%, 100% and 100% ethanol to dehydrate in sequence, 15 min each time. Finally, they were embedded in SPI-Pon 812 (SPI, 0529–77-4, USA). An ultramicrotome (UC, Leica) was used to cut 60 nm ultrathin sections, which were stained with 2% uranyl acetate and lead citrate, subsequently. Sections were examined under a Hitachi electron microscope (Hitachi, HT7800, Japan).

### Western blotting

Proteins were extracted by RIPA lysis buffer (Boster, AR0102, China) supplemented with 1% PMSF (Boster, AR1178, China), 1% protease inhibitor (Boster, AR1182, China) and 1% protease inhibitor cocktail (MCE, HY-K0010, China). SDS-PAGE was used to separate the proteins, which were then transferred to PVDF membranes. After blocking with Tris-buffered saline-Tween with 5% bovine serum albumin for 1 h at room temperature, membranes were incubated with primary antibodies against SLC7A11 (1:500; ABclonal, A13685, China), GPX4 (1:500; ABclonal, A1933, China), ACSL4(1:1000; Affinity Biosciences, DF12141, USA), LPCAT3 (1:500; ABclonal, A17604, China), ALOX15 (1:500; ABclonal, A6864, China) and β-Actin (1:200,000; ABclonal, AC026, China) at 4 °C overnight. Then, the membranes were hybridised with goat anti-rabbit secondary antibody (1:10,000; Boster, BA1055, China) and visualised by ChemiDoc Touch Imaging System (Bio-Rad, USA).

### Reagent

RSL3 is one of ferroptosis agonists. CCSMCs were exposed to 500 nmol/L RSL3 (MCE, HY-100218A, China) for 24 h in order to induce ferroptosis. Ferroptosis inhibitor ferrostatin-1 (Fer-1; 1 μmol/L; MCE, HY-100579, China) was added to medium to inhibit ferroptosis in CCSMCs.

### Statistical analysis

The IHC were quantified by Image-Pro Plus 6.0 software, and results were analysed with GraphPad Prism 8.3. Statistical analyses were conducted using one-way ANOVA followed by the Tukey’s test for post hoc comparisons. *P* < 0.05 was defined as statistically significant.

## Results

### Characterisation of HUCMSCs and CCSMCs

**Fig. 1:**
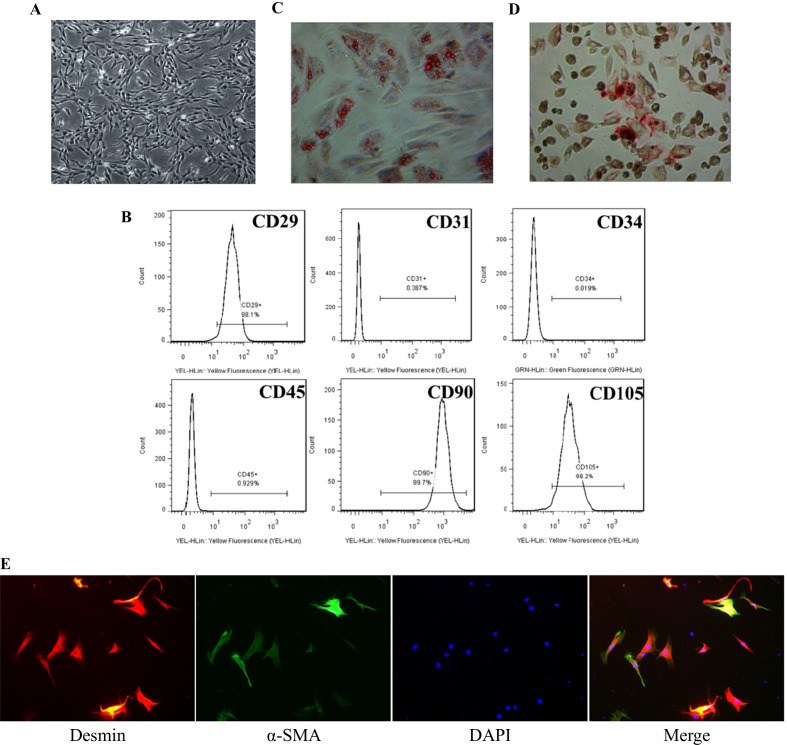
Primary culture and characterisation of HUCMSCs. **A** Morphological features of HUCMSCs at third passage. The magnification is × 100. **B** Representative flow cytometry histograms of HUCMSCs. **C** Adipogenic differentiation of HUCMSCs assessed by Oil Red O staining. The magnification is × 200. **D** Osteogenic differentiation of HUCMSCs assessed by Alizarin Red S staining. The magnification is × 200. **E** Representative immunofluorescence results of CCSMCs show positive expression for α-SMA and desmin. The magnification is × 100. *HUCMSCs* human umbilical cord mesenchymal stem cells, *CCSMCs* corpus cavernosum smooth muscle cells, *α-SMA* α-smooth muscle actin, *DAPI* 4’,6-diamidino-2-phenylindole

The isolated stem cells showed typical fibroblast morphology when they were passed to the third passage (Fig. 1A). To identify HUCMSCs, we used flow cytometry to detect the expression of specific markers. As shown in the Fig. 1B, most cells expressed CD29, CD90, and CD105, although CD31, CD34, and CD45 were seldom expressed. Meanwhile, with the multipotency of HUCMSCs, we successfully induced them into adipocytes and osteoblasts, which indicated by Oil red O staining and Alizarin red S staining, respectively (Fig. 1C, D).

CCSMCs were isolated from rat penis, as in our prior investigation [34]. The cells were then identified by immunofluorescence staining (Fig. 1E), which revealed that they exhibited significant levels of α-SMA and desmin, suggesting that the cells we extracted and cultivated were CCSMCs.

### Metabolic indices

**Fig. 2:**
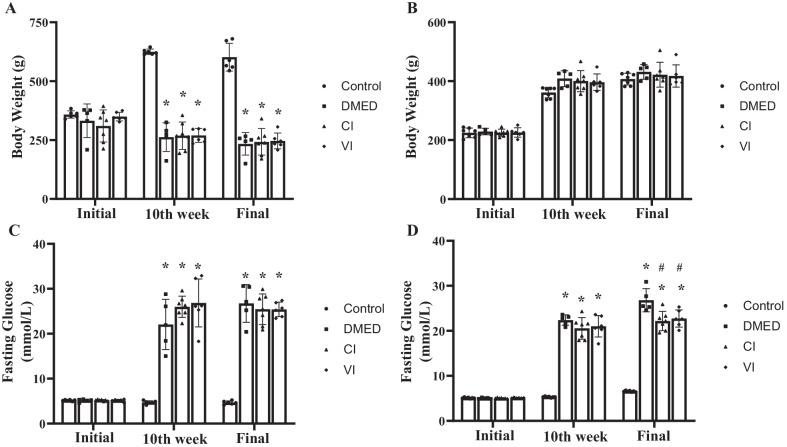
Metabolic status of rats. Body weights of T1DM (**A**) and T2DM (**B**) rats in each group. Fasting glucose of T1DM (**C**) and T2DM (**D**) rats in each group. Data are expressed as means ± standard deviation. *P < 0.05 versus the control group; #*P* < 0.05 versus the DMED group. *Con* control, *DMED* diabetes mellitus erectile dysfunction, *CI* corpus cavernosum injection, *VI* tail vein injection, *T1DM* type 1 diabetes mellitus, *T2DM* type 2 diabetes mellitus

As illustrated in Fig. 2, there was no significant difference in the initial body weight and fasting blood glucose among all groups, whether T1DM or T2DM rats. In T1DM rats, the DMED group, CI group, and VI group had higher blood glucose and lower body weight than the control group ten weeks after induction, but in T2DM rats, the DMED group, CI group, and VI group had higher blood glucose and body weight than the control group. These findings consistently demonstrated that we had successfully created a rat model of T1DM and T2DM.

### HUCMSCs could improve erectile function in DMED rats

**Fig. 3:**
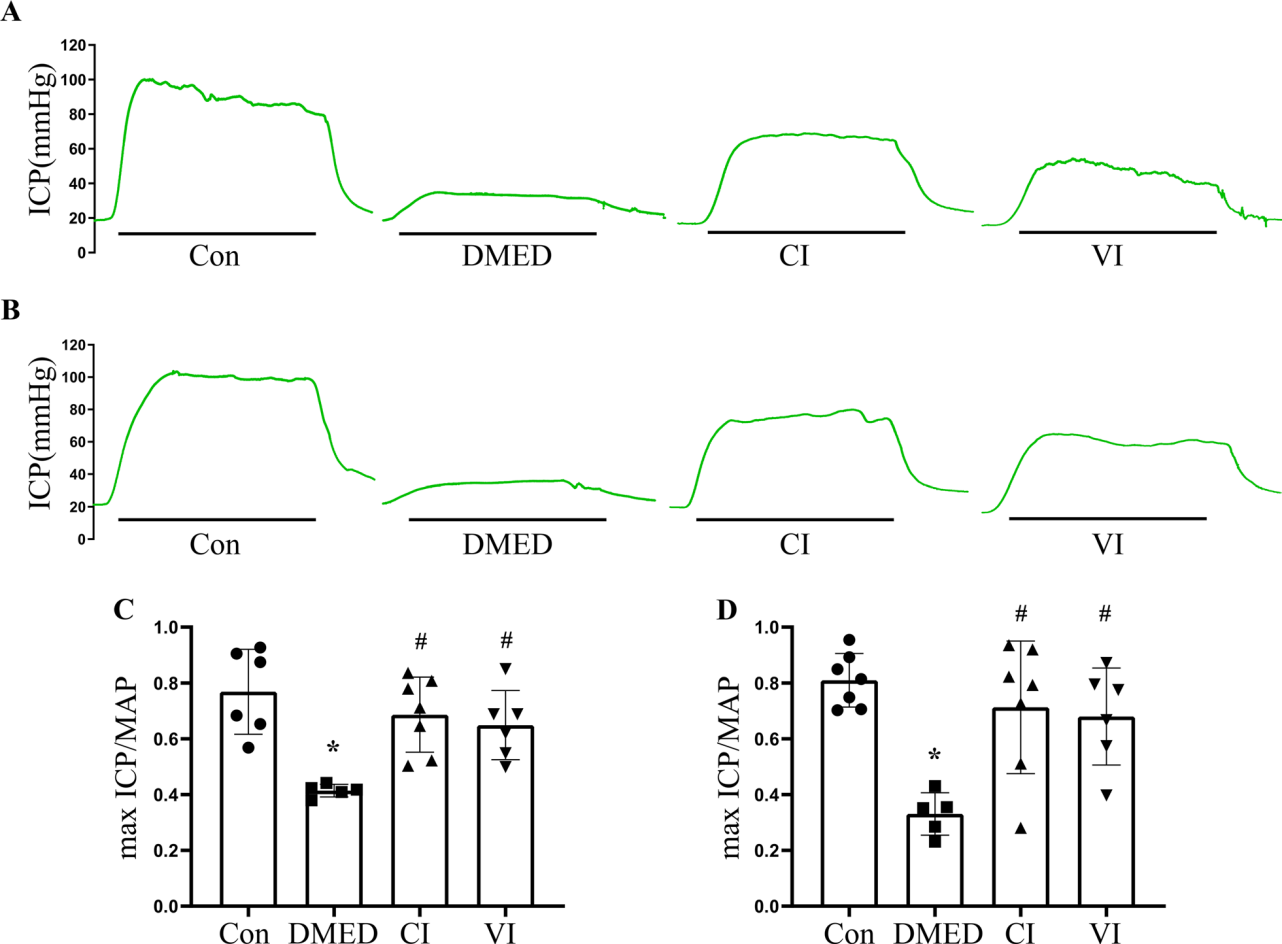
Evaluation of erectile function. **A** Representative ICP traces of T1DM rats (stimulation parameters: 15 Hz; 5.0 V; 1 min). **B** Representative ICP traces of T2DM rats (stimulation parameters: 15 Hz; 5.0 V; 1 min). **C** The max ICP/MAP values of each groups in T1DM rats. **D** The max ICP/MAP values of each groups in T2DM rats. Data are expressed as means ± standard deviation. *P < 0.05 versus the control group; #*P* < 0.05 versus the DMED group. *Con* control, *DMED* diabetes mellitus erectile dysfunction, *CI* corpus cavernosum injection, *VI* tail vein injection, *ICP* intracavernous pressure, *MAP* mean atrial pressure

The ratio of max ICP to MAP reflected erectile function. In T1DM rats (Fig. 3A, C), the indicator was dramatically reduced in the DMED group and significantly improved following injection of HUCMSCs into the corpus cavernosum or tail vein. It also revealed a consistent trend in T2DM rats at the same period (Fig. 3B, D).

### HUCMSCs suppressed penile histological changes in vivo

**Fig. 4:**
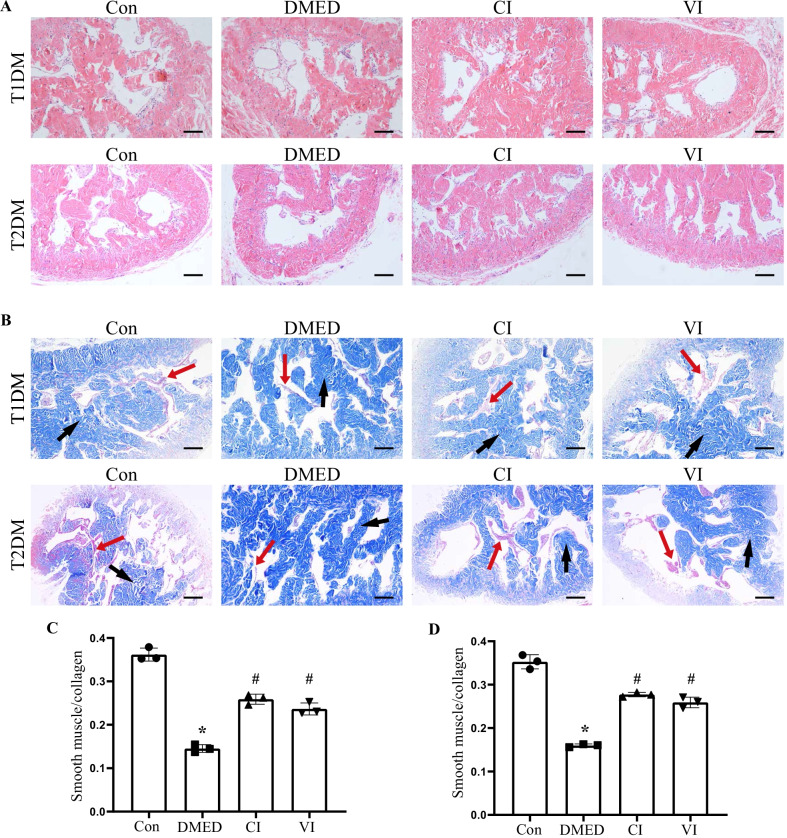
Hematoxylin–eosin staining and Masson’s trichrome staining of penile specimens. **A** Representative images of smooth muscle subjected to hematoxylin–eosin staining. Scale bars = 100 μm. **B** Representative images of smooth muscle stained with Masson’s trichrome staining. The red arrows indicate smooth muscle, and the black arrows indicate collagen fibers. Scale bars = 100 μm. **C** The ratio of smooth muscle to collagen contents in penile section of T1DM rats. **D** The ratio of smooth muscle to collagen contents in penile section of T2DM rats. Data are expressed as means ± standard deviation. *P < 0.05 versus the control group; #*P* < 0.05 versus the DMED group. *T1DM* type 1 diabetes mellitus, *T2DM* type 2 diabetes mellitus, *Con* control, *DMED* diabetes mellitus erectile dysfunction, *CI* corpus cavernosum injection, *VI* tail vein injection

We detected the colonisation of stem cells by EdU assay (Additional file 1: Fig. S1), however only very low fluorescence intensity was seen in the penile tissue of type 1 and type 2 DMED rats following stem cell injection. The corpus cavernosum smooth muscle atrophy was discovered in the diabetic group (Fig. 4B). The smooth muscle regeneration and reduction in cavernous fibrosis, on the other hand, occurred in the CI and VI groups, and the smooth muscle/collagen ratio rose considerably in both groups (Fig. 4C, D). Importantly, following HUCMSC injection, there was no substantial alteration in the anatomy of the rat penis, no inflammation, no tissue necrosis, and no tumour growth in the corpus cavernosum (Fig. 4A).

### HUCMSCs retained NO activity in vivo

We employed particular kits to quantify NO and cGMP concentrations in rat penises, same as we did in our previous work [38]. NO and cGMP production in the penis of DMED rats were severely suppressed, although they were somewhat enhanced after stem cell injection, according to the findings (Additional file 2: Fig. S2A–D). We next used immunohistochemistry to examine the quantity of eNOS and nNOS in the rat penis, two enzymes that mediated the synthesis of NO in the corpus cavernosum for erection maintenance. The levels of eNOS and nNOS in the DMED group were found to be lower than those in the control group, whereas they were somewhat higher in the CI and VI groups (Additional file 2: Fig. S2E, F).

### HUCMSCs reduced iron content and inhibited oxidative stress in vivo

**Fig. 5:**
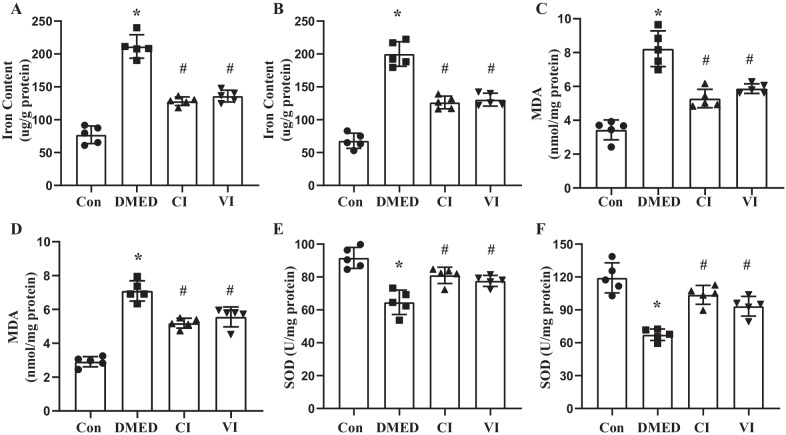
Assessment of iron content and oxidative activity. Iron contents in the corpus cavernosum of T1DM (**A**) and T2DM (**B**) rats. Levels of MDA in the corpus cavernosum of T1DM (**C**) and T2DM (**D**) rats. Levels of SOD in the corpus cavernosum of T1DM (**E**) and T2DM (**F**) rats. Data are expressed as means ± standard deviation. *P < 0.05 versus the control group; #*P* < 0.05 versus the DMED group. *T1DM* type 1 diabetes mellitus, *T2DM* type 2 diabetes mellitus, *Con* control, *DMED* diabetes mellitus erectile dysfunction, *CI* corpus cavernosum injection, *VI* tail vein injection, *MDA* malondialdehyde, *SOD* superoxide dismutase

**Fig. 6:**
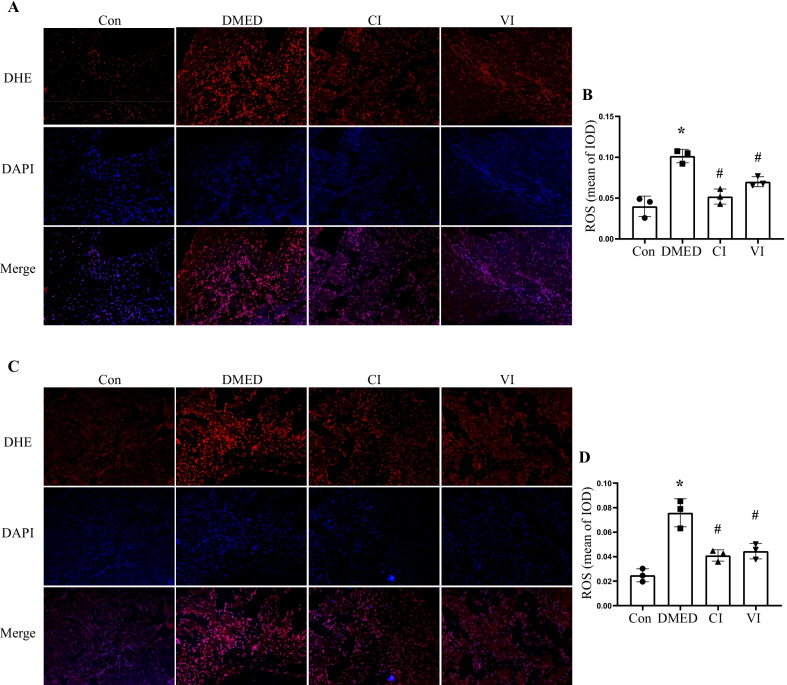
Effects of HUCMSCs on reducing ROS production in vivo. Representative immunofluorescence (× 200) (**A**) and quantification (**B**) of ROS in the corpus cavernosum of T1DM rats. Representative immunofluorescence (× 200) (**C**) and quantification (**D**) of ROS in the corpus cavernosum of T2DM rats. Data are expressed as means ± standard deviation. *P < 0.05 versus the control group; #*P* < 0.05 versus the DMED group. *ROS* reactive oxygen species, *IOD* integrated option density, *T1DM* type 1 diabetes mellitus, *T2DM* type 2 diabetes mellitus, *Con* control, *DMED* diabetes mellitus erectile dysfunction, *CI* corpus cavernosum injection, *VI* tail vein injection, *DAPI* 4’,6-diamidino-2-phenylindole

To explore and confirm the role of ferroptosis in DMED, we examined the iron content and oxidative stress in rat penis. The results revealed that the iron content in DMED rats' penis was much higher, but the iron content of rats in the CI and VI groups was significantly lower, which was observed in both T1DM and T2DM rats (Fig. 5A, B). MDA levels exhibited a similar trend as a sign of oxidative stress, and HUCMSCs obviously reduced MDA concentration in DMED rats (Fig. 5C, D). In contrast, SOD acted as an antioxidant in the body and exhibited the exact opposite changes to MDA (Fig. 5E, F). Then, DHE staining was conducted in the corpus cavernosum to reflect ROS (Fig. 6). The results showed that ROS expression was higher in the DMED group, and HUCMSCs attenuated ROS production significantly in the CI and VI groups.

### HUCMSCs decreased oxidative stress in vitro

**Fig. 7:**
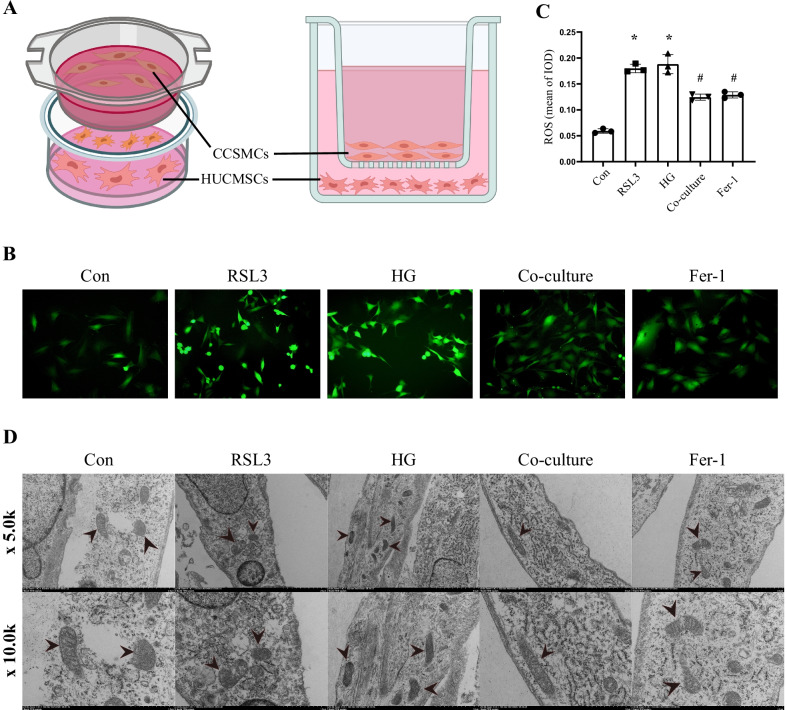
HUCMSCs attenuated ROS production and mitochondrial aberrations in vitro. **A** The diagram of the coculture system of HUCMSCs and CCSMCs. Representative immunofluorescence (× 200) (**B**) and quantification (**C**) of ROS in CCSMCs of each group. (**D**) The morphology of the mitochondria in CCSMCs analyzed by TEM. Original magnification × 5.0 k and × 10.0 k. Data are expressed as means ± standard deviation. *P < 0.05 versus the control group; #*P* < 0.05 versus the HG group. *HUCMSCs* human umbilical cord mesenchymal stem cells, *CCSMCs* corpus cavernosum smooth muscle cells, *Con* control, *HG* high glucose, *Fer-1* ferrostatin-1, *ROS* reactive oxygen species, *IOD* integrated option density, *TEM* transmission electron microscope

In order to confirm the inhibitory effect of HUCMSCs on oxidative stress in vitro. We cultivated HUCMSCs and CCSMCs in the co-culture system (Fig. 7A). The amount of ROS was measured by DCFH-DA staining (Fig. 7B, C). The findings demonstrated that smooth muscle cells produced more ROS than usual in a high-glucose environment and after RSL3 treatment, and that the quantity of ROS dropped dramatically after co-culture with HUCMSCs, but CCSMCs exposed to the ferroptosis inhibitor Fer-1 showed a consistent trend.

### HUCMSCs attenuated ferroptosis through SLC7A11/GPX4/ACSL4 pathway in vitro

Mitochondria in ferroptotic cells exhibited specific morphological changes. We observed the shrunken mitochondria, decreased or complete absence of mitochondria cristae and high electron density in RSL3-treated CCSMCs by electron microscopy. Meanwhile, CCSMCs treated with high glucose displayed comparable alterations, suggesting that diabetes might cause ferroptosis in CCSMCs. However, after co-culturing with HUCMSCs, the morphological changes in mitochondria were significantly improved, and normal mitochondrial structure could be observed (Fig. 7D).

**Fig. 8:**
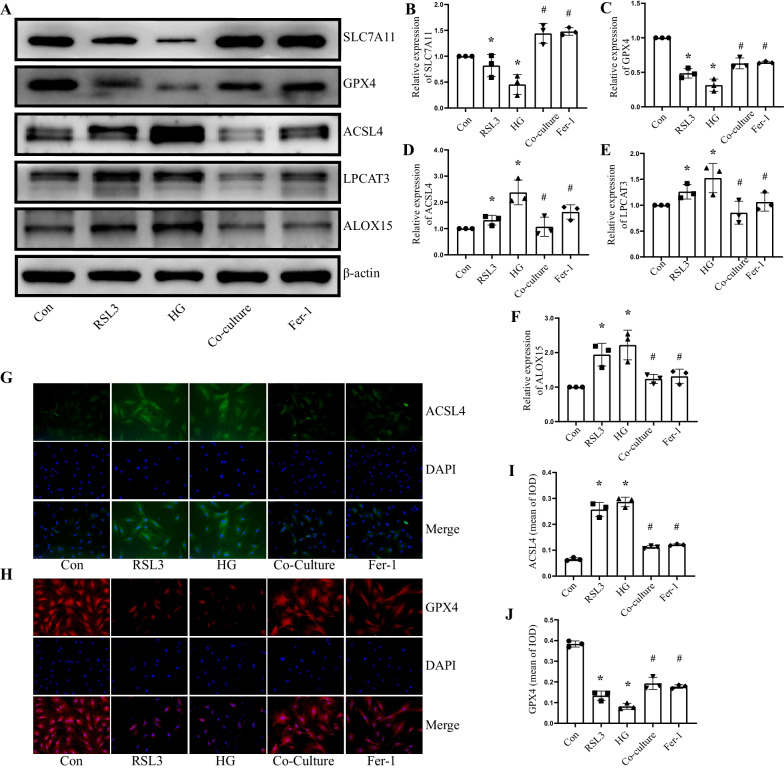
HUCMSCs attenuated ferroptosis through the SLC7A11/GPX4/ACSL4 pathway in vitro. Representative immunoblot (**A**) and quantification (**B**–**F**) of SLC7A11, GPX4, ACSL4, LPCAT3 and ALOX15 in CCSMCs of each group. Representative immunofluorescence (× 200) (**G**) and quantification (**I**) of ACSL4 in CCSMCs of each group. Representative immunofluorescence (× 200) (**H**) and quantification (**J**) of GPX4 in CCSMCs of each group. Data are expressed as means ± standard deviation. *P < 0.05 versus the control group; #*P* < 0.05 versus the HG group. *Con* control, *HG* high glucose, *Fer-1* ferrostatin-1, *IOD* integrated option density, *DAPI* 4’,6-diamidino-2-phenylindole

As an important component of the cystine transporter system Xc^**−**^, SLC7A11-mediated cystine uptake plays a vital role in inhibiting cellular oxidative stress. In Fig. 8, the expression of SLC7A11 in CCSMCs treated with high glucose and RSL3 was significantly lower than in normal cells, according to Western blot analysis, and HUCMSCs had a clear ability to rescue its expression, which was consistent with CCSMCs treated with Fer-1. GPX4 is one of the most well-known ferroptosis defence pathways, and its expression in each group followed the same pattern as SLC7A11. While ACSL4, LPCAT3, and ALOX15, which promote oxidative stress during ferroptosis, were clearly elevated in CCSMCs treated with high glucose, they were significantly reduced after co-culture with HUCMSCs.

## Discussion

It's been reported that the global incidence of erectile dysfunction is expected to rise to 322 million cases by 2025 [1, 39, 40]. Diabetes is a major risk factor for erectile dysfunction, and peripheral neuropathy, atherosclerosis of large vessels, endothelial dysfunction of small arteries and hypogonadism can eventually lead to erectile dysfunction [41–44]. We investigated the effect of HUCMSCs on DMED rats' erectile function through corpus cavernosum injection and tail vein injection, respectively, based on the construction of T1DM and T2DM ED rats. The impaired erectile function of DMED rats was significantly improved without side effects, and the underlying molecular mechanism by which HUCMSCs could attenuate ferroptosis in CCSMCs was confirmed, all of which indicated that HUCMSCs could be a promising emerging therapeutic strategy for DMED.

HUCMSCs, a kind of naïve stem cell found in the human body, may be collected in great quantities without posing a risk to the donor, and the application of HUCMSCs does not raise ethical problems. In recent years, some studies have revealed that bone mesenchymal stem cells (BMSCs) and adipose-derived stem cells (ADSCs) could improve the erectile function of DMED to some extent [45, 46], similar to how HUCMSCs have been shown to have good therapeutic effects on diseases of the immune system, cardiovascular system, and digestive system [47–49]. The real therapeutic benefits of BMSCs and ADSCs to DMED, however, are restricted due to their high degree of differentiation and low biological stability [50–54]. Furthermore, certain clinical trials have showed that PDE5i is still necessary to sustain erection following the administration of BMSCs or ADSCs [55, 56]. T1DM and T2DM rats given HUCMSCs had considerable improvement in their erectile function, and there were no evident rejection or other adverse effects following injection, demonstrating the biological safety of HUCMSCs. The favourable impact of stem cells in tissue repair, according to Matz et al., is more dependent on paracrine activity, the production of cytokines and growth factors that reduce inflammation and promote healing, than on colonisation and differentiation [57]. We labelled the cells with EdU before injecting them to investigate the exact mechanism of HUCMSCs in the treatment of DMED [58–60]. It revealed that only a small number of labelled HUCMSCs colonised the rat penis. Since HUCMSCs are human-derived cells, it is difficult for HUCMSCs to directly differentiate into various cell types in the rat penis to repair tissues, which might be the reason why we observed only a small number of stem cell colonisation. Päth et al. also disclosed that, despite not having successfully differentiated into β cells, mesenchymal stem cells (MSCs) can secrete a variety of immunomodulatory and tissue regeneration factors to lower blood glucose levels [61]. In several clinical trials, researchers discovered that MSCs reduced and delayed the destruction of β cells in many T1DM patients, but did not restore their function [62–64]. And the anti-inflammatory properties of MSCs were exploited to treat chronic low-grade inflammation in T2DM patients, which was thought to be a major cause of insulin resistance and β-cell dysfunction [65]. Based on the above, we speculated that HUCMSCs might primarily repair tissue via paracrine. Interestingly, we noticed that there was a slight improvement in hyperglycemia in T2DM ED rats after injection, but not in T1DM ED rats, even though HUCMSCs did not restore fasting glucose to normal in both types of DMED rats. Based on these, we preliminarily confirmed that HUCMSCs ameliorated ED through a glucose independent manner. On the other hand, more severe hyperglycemia might exacerbate irreversible damage to pancreatic cells, vascular smooth muscle cells, vascular endothelial cells, and cavernous nerves in T1DM rats.

Stem cell treatment for ED was previously administered mostly through the corpus cavernosum, abdominal cavity, and tail vein. There was only one study comparing the efficacy of corpus cavernosum and tail vein injection of ADSCs in rats with nerve-injured ED [66]. In fact, DMED is currently more common than nerve-injured ED. Our findings are the first to compare the therapeutic effects of HUCMSCs on two types of DMED rats by CI and VI. The therapeutic effects of the two methods are almost identical, and there are no obvious differences in various indicators. It was demonstrated that HUCMSCs could effectively improve the erectile function of rats through CI and VI. This could serve as a starting point for future research on HUCMSCs as a DMED treatment.

As revealed by Masson's trichrome staining, we found that the ability of HUCMSCs to suppress cavernous fibrosis might contribute to their therapeutic impact in ED. One of the key causes of DMED, as revealed in a previous study [67, 68], is corpus cavernosum fibrosis, which is induced by reduced corpus cavernosum smooth muscle and increased collagen. In both types of DMED rats, we noticed a considerable loss in smooth muscle and a significant rise in collagen, but these histological alterations were greatly relieved following stem cell therapy, indicating that HUCMSCs had a good anti-fibrotic activity.

NO causes the corpus cavernosum smooth muscle to relax by promoting the synthesis of cGMP in CCSMCs, which is produced by eNOS in cavernous endothelial cells and nNOS in cavernous neurons. Our data argued that HUCMSCs might increase the abundance of eNOS and nNOS in the corpus cavernosum of DMED rats, boosting NO and cGMP levels, implying that HUCMSCs could alleviate DMED via the NO/cGMP pathway.

Ferroptosis plays a crucial role in the occurrence and progression of various diseases, and regulating the ferroptosis pathway presents a potential therapeutic strategy for many diseases [69–71]. The intracellular antioxidant system, System Xc^**−**^, is made up of two subunits, SLC7A11 and SLC3A2. Through the System Xc^**−**^, cysteine and glutamic acid collaborate to synthesise glutathione, and glutathione then lowers ROS and reactive nitrogen via glutathione peroxidase (GPX). Inhibition of System Xc^**−**^ function interferes with GSH production, resulting in reduced GPX activity, decreased cellular antioxidant capacity, lipid ROS buildup, and, ultimately, oxidative damage and ferroptosis [72]. GPX4, member of the GPX family, is a critical regulator of ferroptosis via limiting the formation of lipid peroxides. GPX4 converts glutathione to oxidised glutathione peptide (GSSG), and reduce cytotoxic lipid peroxidation to the corresponding alcohol. Inhibition of GPX4 activity leads to accumulation of lipid peroxides, a hallmark of ferroptosis [73]. However, the role of ferroptosis in ED remains unclear. First, we found that HUCMSCs diminished iron content and oxidative stress levels in DMED rats' penis, including MDA, SOD, and ROS. Second, HUCMSCs were able to enhance the abundance of SLC7A11 and GPX4 while decreasing the expression of lipid metabolism-related genes such ACSL4, LPCAT3, and ALOX15. Furthermore, intracellular mitochondria treated with high glucose showed morphological abnormalities that were rescued by HUCMSCs. Those proved that HUCMSCs might alleviate DMED by prohibiting the ferroptosis signalling pathway in CCSMCs.

There are some limitations in current study. We utilised the DMED rat model to explore the impact of HUCMSCs on DMED and the underlying mechanism, however additional study is needed to better understand its safety, effectiveness, and other unknown concerns before it can be used in clinical practise. In the following investigation, we'll build rabbit, dog, and possibly monkey animal models to further confirm the therapeutic efficacy of HUCMSCs on DMED. Second, the injection dose used in the study was one million cells, which is consistent with other studies [50, 51, 74–76]. It is unclear whether there is an ideal dose yet, therefore we will compare outcomes from different doses in future studies to find the optimal injection dose. Finally, HUCMSCs secrete cytokines [77], chemokines [78], growth factors [79], proteases [80], and extracellular vesicles [81] to exert paracrine effects, but we have not yet figured out which paracrine mode of HUCMSCs mediate to suppress ferroptosis in CCSMCs. We will conduct appropriate in vivo and in vitro experiments to explore the specific paracrine mode of HUCMSCs repairing CCSMCs, so as to further clarify the mechanism of HUCMSCs in the treatment of DMED.

## Conclusions

In summary, the current investigation found that HUCMSCs improved erectile function in T1DM and T2DM ED rats without major adverse events. Moreover, it was uncovered that HUCMSCs not only inhibited corpus cavernosum fibrosis and activated NOS, but prevented diabetes-induced ferroptosis in CCSMCs. Furthermore, we found that HUCMSCs possessed equal efficacy in the therapy of DMED when injected into the corpus cavernosum and tail vein. These findings provide preclinical evidence for a potential treatment for DMED and new insights into the therapeutic mechanism of HUCMSCs in the treatment of DMED.

## Supplementary Information

**Additional file 1: Fig. S1.** Examination of colonization of HUCMSCs. Representative immunofluorescence (× 200) (**A**) of EdU in the corpus cavernosum of T1DM rats after HUCMSCs injection. Representative immunofluorescence (×200) (**B**) of EdU in the corpus cavernosum of T2DM rats after HUCMSCs injection. *CI* corpus cavernosum injection, *VI* tail vein injection, *EdU* 5-ethynyl-2’-deoxyuridine, *DAPI* 4’,6-diamidino-2-phenylindole

**Additional file 2: Fig. S2.** Assessment of NO and cGMP. The levels of NO in the corpus cavernosum of T1DM (**A**) and T2DM (**B**) rats. The cGMP concentration in the corpus cavernosum of T1DM (**C**) and T2DM (**D**) rats. Representative results of immunohistochemistry analysis of nNOS (**E**) and eNOS (**F**) in the corpus cavernosum of rats. The magnification is × 200. The arrows indicate nNOS and eNOS expression (Because the expression levels of nNOS and eNOS in DMED group were very low, the coloring was very light). (**G**) The relative expression of nNOS and eNOS in the corpus cavernosum of T1DM and T2DM rats. Data are expressed as means ± standard deviation. *P < 0.05 versus the control group; #*P* < 0.05 versus the DMED group. *cGMP* cyclic guanosine monophosphate, *NO* nitric oxide, *T1DM* type 1 diabetes mellitus, *T2DM* type 2 diabetes mellitus, *Con* control, *DMED* diabetes mellitus erectile dysfunction, *CI* corpus cavernosum injection, *VI* tail vein injection, *eNOS* endothelial nitric oxide synthase, *nNOS* neuronal nitric oxide synthase

## Data Availability

The data that support the findings of this study are available from the corresponding author upon reasonable request.

## Abbreviations

ED: Erectile dysfunction
DMED: Diabetic mellitus erectile dysfunction
HUCMSCs: Human umbilical cord mesenchymal stem cells
T1DM: Type 1 diabetes mellitus
T2DM: Type 2 diabetes mellitus
CCSMCs: Corpus cavernosum smooth muscle cells
DM: Diabetes mellitus
MHC: Major histocompatibility complex
DMED: Dulbecco’s modified Eagle’s medium
SD: Sprague–Dawley
ZDF: Zucker Diabetic Fatty
APO: Apomorphine
CI: Corpus cavernosum injection
VI: Tail vein injection
EdU: 5-Ethynyl-2’-deoxyuridine
ICP: Intracavernous pressure
MAP: Mean arterial pressure
H&E: Hematoxylin–eosin
eNOS: Endothelial nitric oxide synthase
nNOS: Neuronal nitric oxide synthase
DAPI: 4', 6-Diamidino-2-phenylindole
MDA: Malondialdehyde
SOD: Superoxide dismutase
cGMP: Cyclic guanosine monophosphate
NO: Nitric oxide
TEM: Transmission electron microscope
BMSCs: Bone mesenchymal stem cells
ADSCs: Adipose-derived stem cells
MSCs: Mesenchymal stem cells
GPX: Glutathione peroxidase
GSSG: Glutathione peptide
