# Supplementary figures and images for: Human umbilical cord mesenchymal stem cells ameliorate erectile dysfunction in rats with diabetes mellitus through the attenuation of ferroptosis
Source: Stem Cell Res Ther. 2022 Sep 5;13:450. doi: 10.1186/s13287-022-03147-w (PMC9444126; doi:10.1186/s13287-022-03147-w)

**A**

EdU

DAPI

Merge

CI

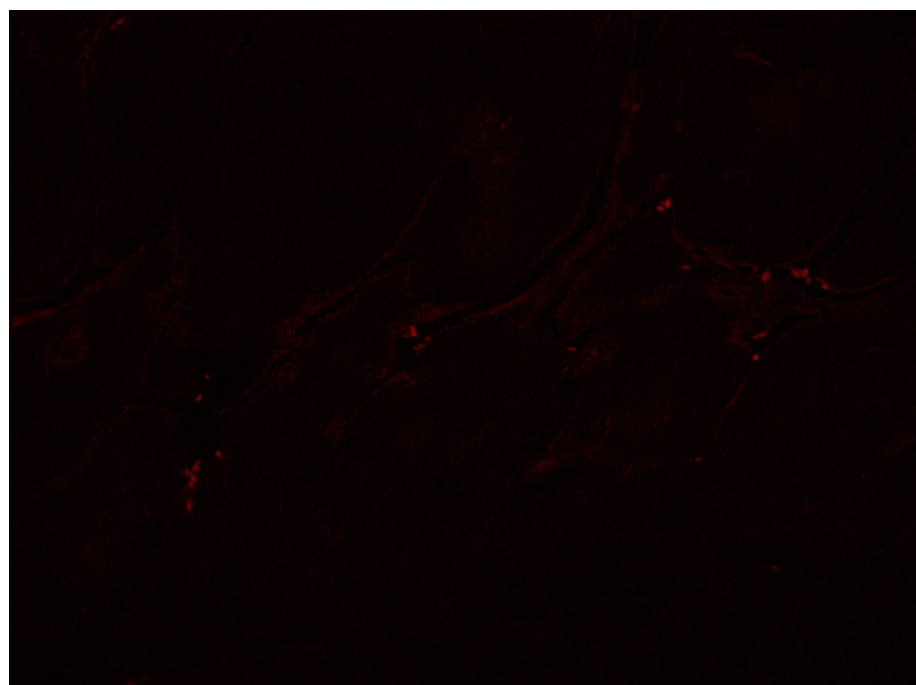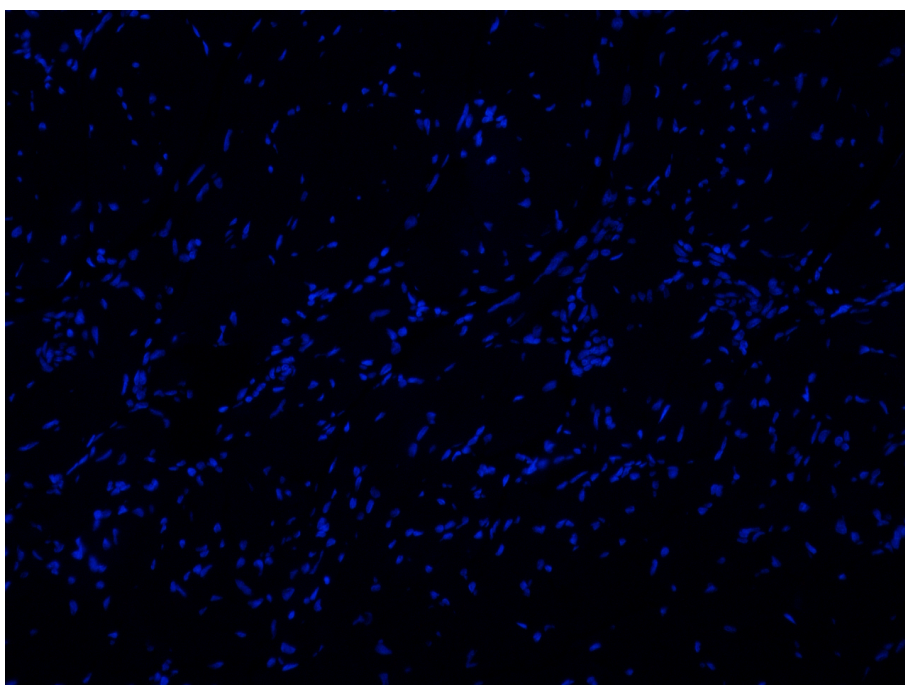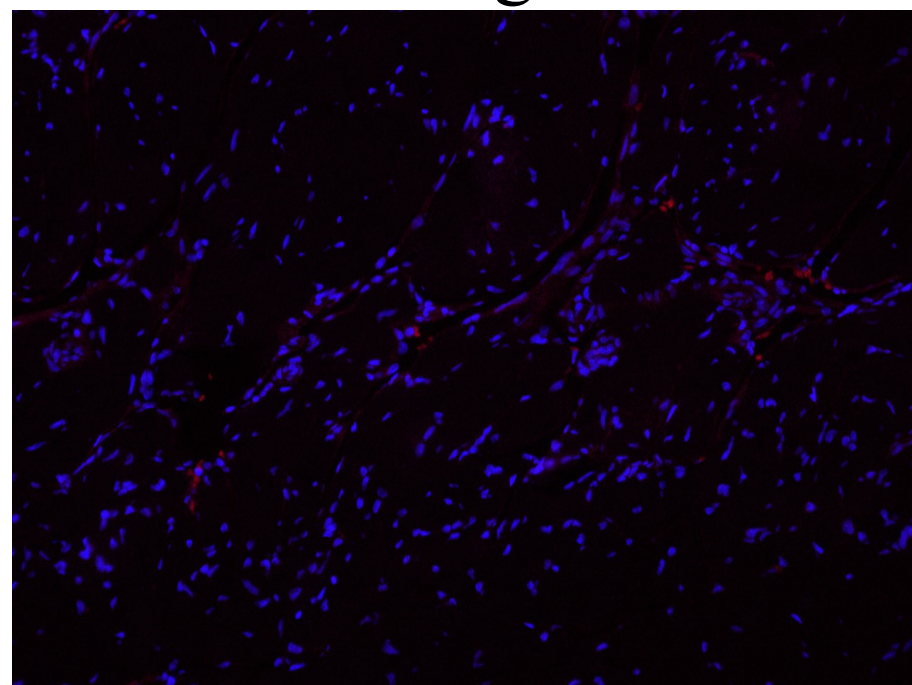

VI

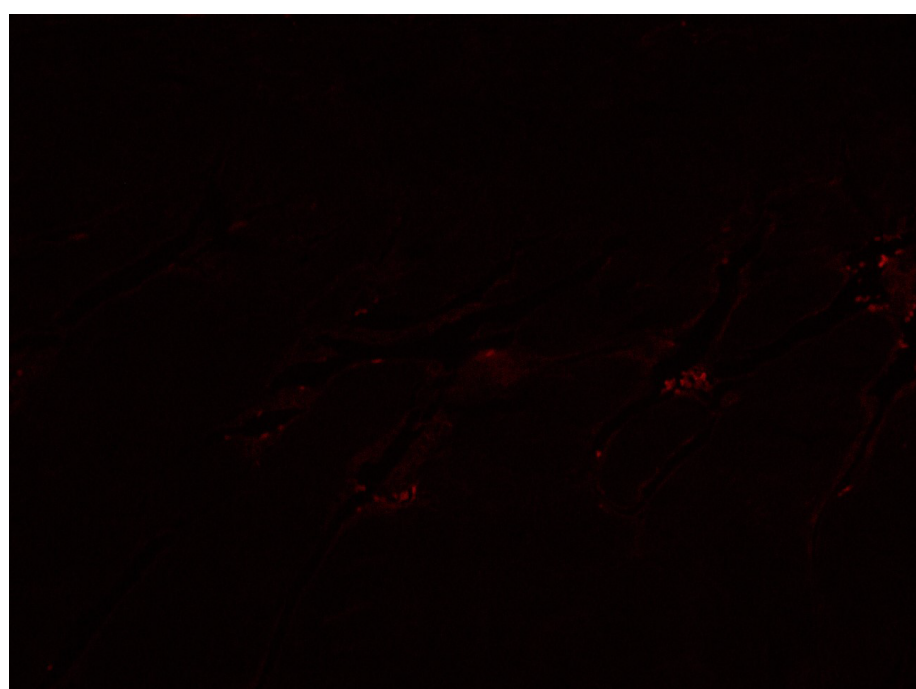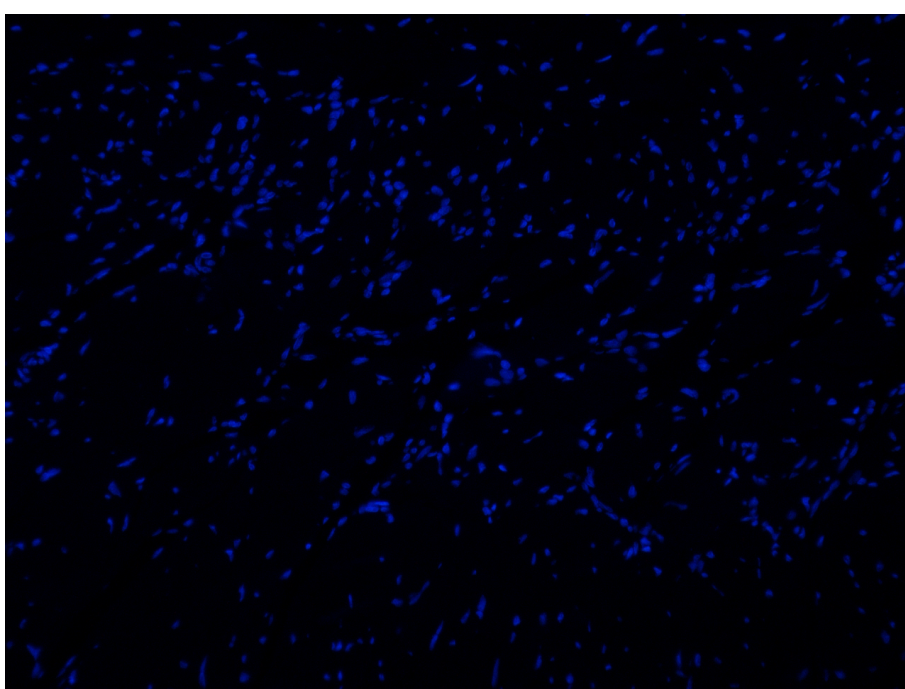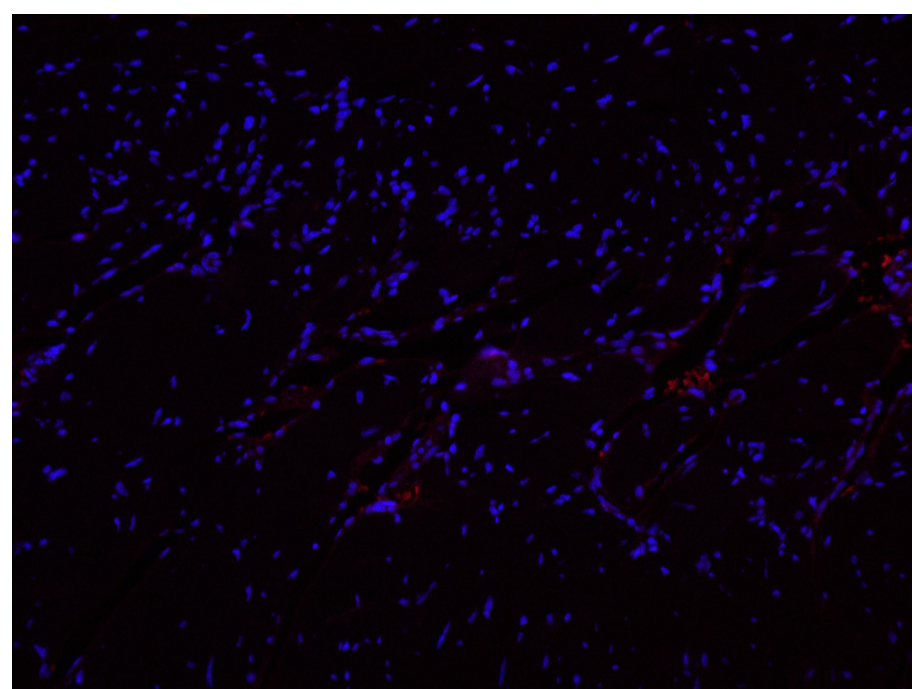

**B**

EdU

DAPI

Merge

CI

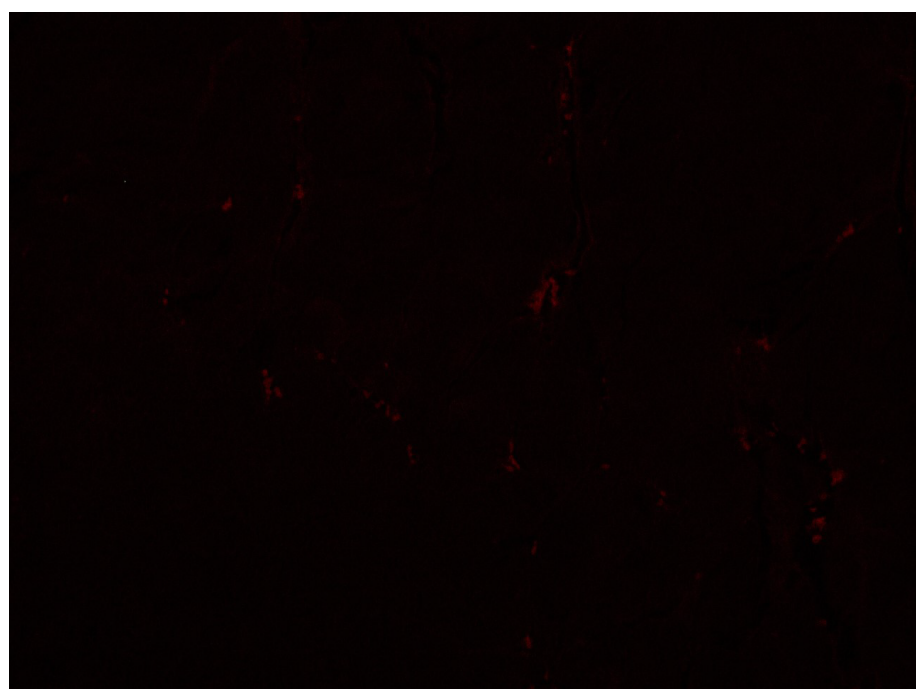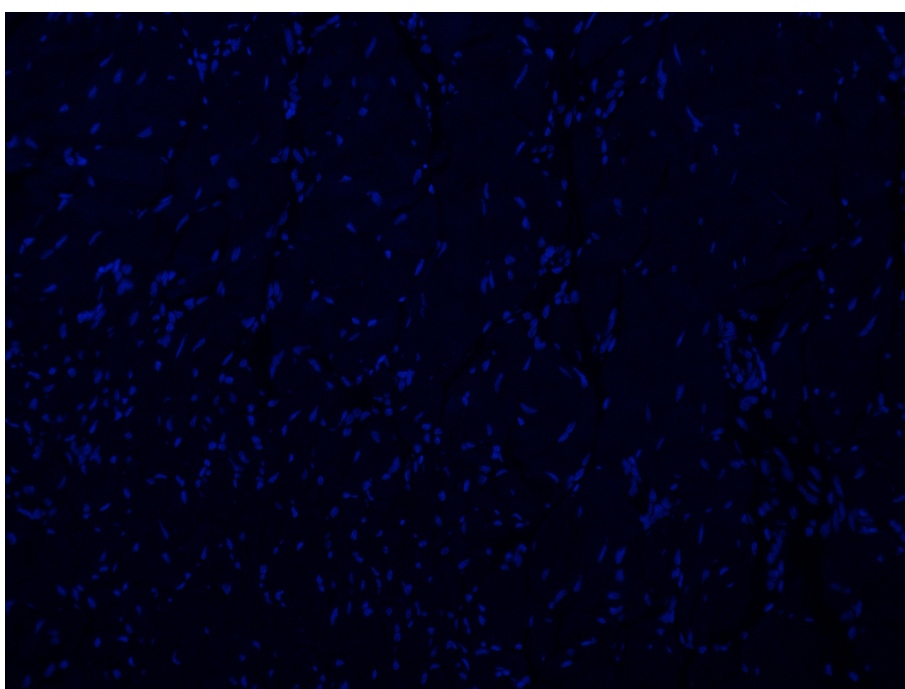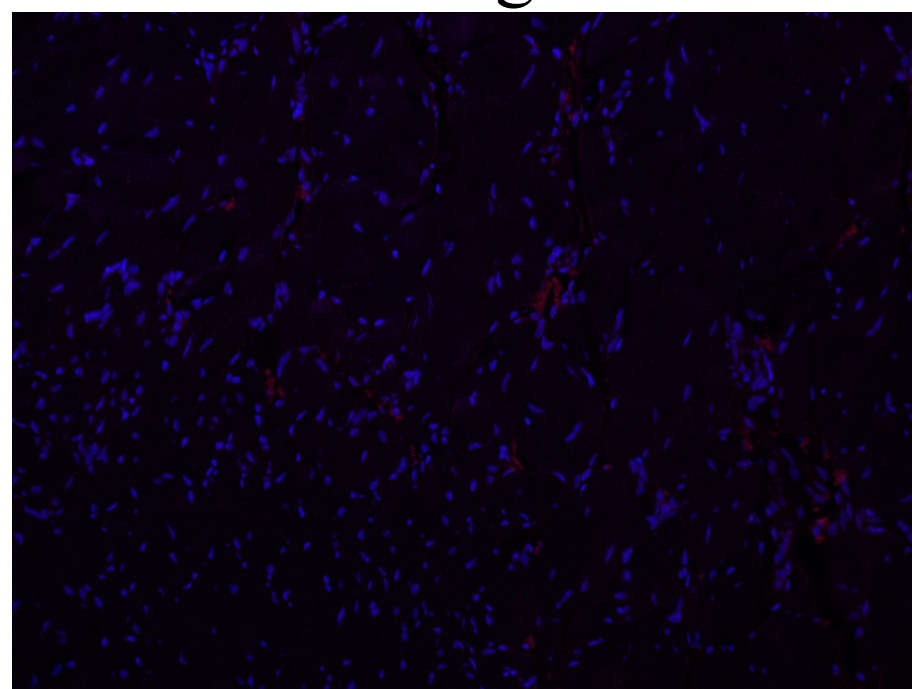

VI

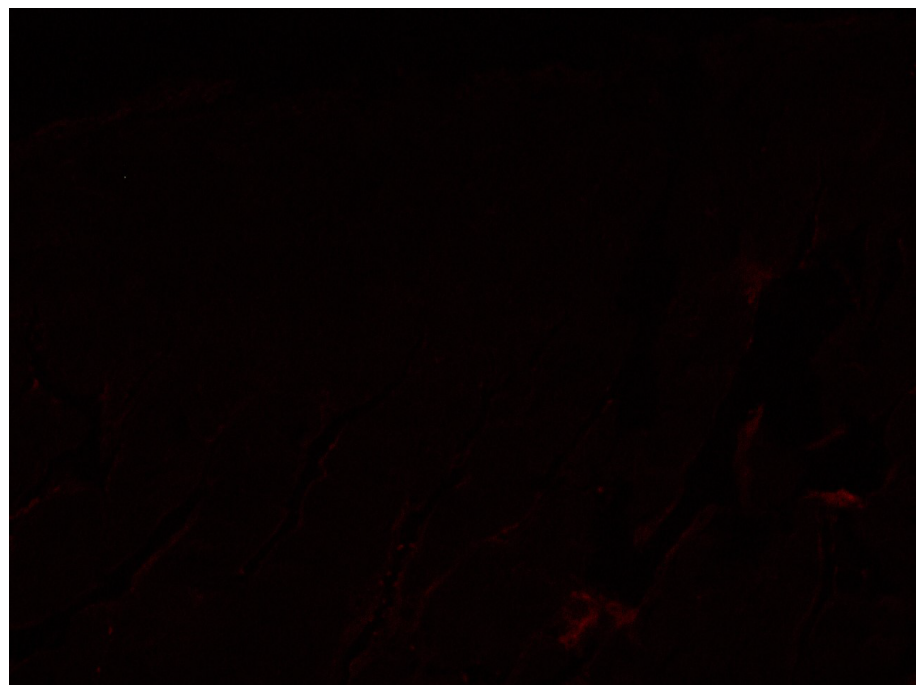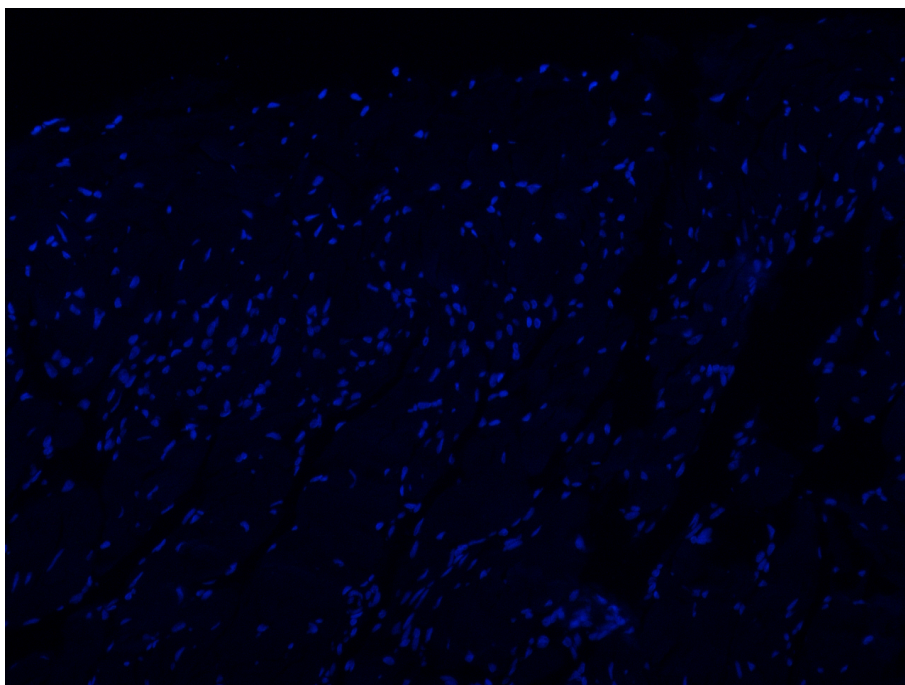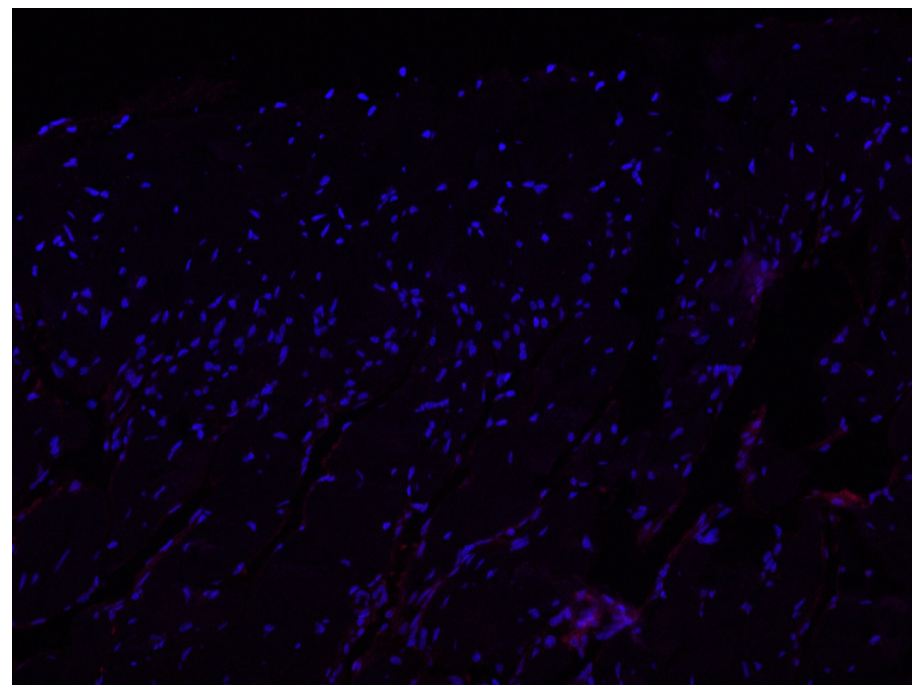

Supplement: Supplementary file 1 — Additional file 1: Fig. S1. Examination of colonization of HUCMSCs. Representative immunofluorescence (× 200) (A) of EdU in the corpus cavernosum of T1DM rats after HUCMSCs injection. Representative immunofluorescence (×200) (B) of EdU in the corpus cavernosum of T2DM rats after HUCMSCs injection. CI corpus cavernosum injection, VI tail vein injection, EdU 5-ethynyl-2’-deoxyuridine, DAPI 4’,6-diamidino-2-phenylindole [file 13287_2022_3147_MOESM1_ESM.pdf]

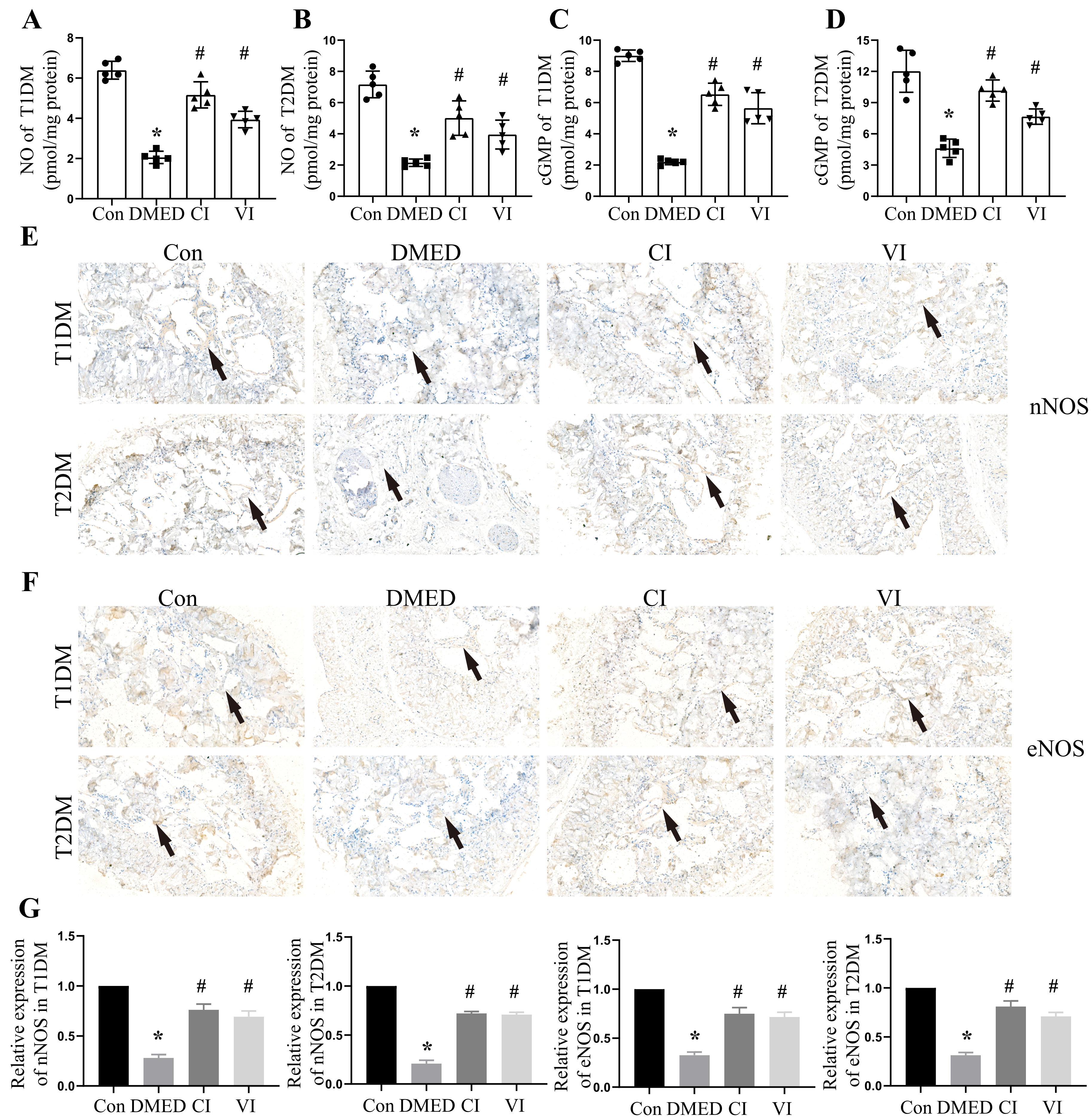

Supplement: Supplementary file 2 — Additional file 2: Fig. S2. Assessment of NO and cGMP. The levels of NO in the corpus cavernosum of T1DM (A) and T2DM (B) rats. The cGMP concentration in the corpus cavernosum of T1DM (C) and T2DM (D) rats. Representative results of immunohistochemistry analysis of nNOS (E) and eNOS (F) in the corpus cavernosum of rats. The magnification is × 200. The arrows indicate nNOS and eNOS expression (Because the expression levels of nNOS and eNOS in DMED group were very low, the coloring was very light). (G) The relative expression of nNOS and eNOS in the corpus cavernosum of T1DM and T2DM rats. Data are expressed as means ± standard deviation. *P < 0.05 versus the control group; #P < 0.05 versus the DMED group. cGMP cyclic guanosine monophosphate, NO nitric oxide, T1DM type 1 diabetes mellitus, T2DM type 2 diabetes mellitus, Con control, DMED diabetes mellitus erectile dysfunction, CI corpus cavernosum injection, VI tail vein injection, eNOS endothelial nitric oxide synthase, nNOS neuronal nitric oxide synthase [file 13287_2022_3147_MOESM2_ESM.pdf]
